# Editorial: Poultry feeding, nutrition, and quality in the post-antibiotic era

**DOI:** 10.3389/fvets.2024.1386278

**Published:** 2024-03-11

**Authors:** Abdel-Moneim Eid Abdel-Moneim, Ilaria Biasato, Noura M. Mesalam, Abdelrazeq M. Shehata

**Affiliations:** ^1^Department of Biological Applications, Nuclear Research Center, Egyptian Atomic Energy Authority, Cairo, Egypt; ^2^Department of Agricultural, Forest and Food Sciences, University of Turin, Turin, Italy; ^3^Department of Animal Production, Faculty of Agriculture, Al-Azhar University, Cairo, Egypt

**Keywords:** antibiotic alternatives, probiotics, Chinese herbal medicine, plant-derived polysaccharides, direct-fed microbes, phytochemicals, spray-dried plasma powder, poultry

For the majority of the past 50 years, farmers have employed antibiotics to enhance the growth of their livestock. The utilization of antibiotic growth promoters (AGPs) has proven beneficial for animals in terms of their health and performance. However, the presence of AGPs in human-consumed meat may have an impact on normal human immunological function and metabolism. The extensive use of subtherapeutic antibiotics, however, has resulted in the emergence of antibiotic-resistant bacteria. Consequently, the prohibition of AGPs in chicken farms has become a top priority. Nevertheless, removing AGPs from chicken feed represents a significant and impactful change with far-reaching consequences. Poultry farmers require assistance in navigating this transition as they grapple with the challenges of reducing antibiotic usage (1, 2). Therefore, there is an urgent need for innovative solutions to address the aforementioned challenges and enhance the productivity of broiler chickens through the utilization of antibiotic alternatives and unconventional nutritional strategies.

The objective of this Research Topic is to compile articles that can enhance our understanding of the use of non-traditional nutritional techniques and the incorporation of eco-friendly feed additives. These efforts aim to improve poultry welfare and foster sustainable production practices.

Gómez-Verduzco et al. identified in feeding spray-dried plasma (SDP) a promising strategy to counteract mycotoxicosis in broiler chickens. The authors observed improved growth performance (in terms of weight gain and feed efficiency) and immune response (in terms of higher thymus relative weight, increased serum levels of macrophage colony-stimulating factor, and higher IgA concentrations in gut and tracheal washes) when birds were supplemented with 2% SPD.

Plant-derived bioactives and herbal extracts show great potential as environmentally friendly feed additives that can serve as alternatives to antibiotics (3–5). Numerous investigations have demonstrated that including anthocyanin plant extract in laying hen diets can enhance both the production and quality of eggs. Li, Zhou et al. examined the impact of anthocyanin-rich purple corn extract on the performance, antioxidant capacity, egg quality, and amino acid and fatty acid profiles of laying hens in the late stages of egg production. The researchers discovered that consuming purple corn extract, which is high in anthocyanins, can increase the antioxidant capacity of the blood, promote egg production, and elevate the levels of amino acids and fatty acids in hen eggs during the late stages of laying. Another study was conducted by Yao et al. to investigate the impact of supplementing sea buckthorn extract on the production performance, serum biochemical metrics, egg quality, and cholesterol deposition in laying ducks. The researchers realized that incorporating sea buckthorn extract into diets can enhance nutrient utilization, enhance egg weight, optimize egg quality and amino acid composition in eggs, decrease blood lipids, improve the fatty acid profile and yolk cholesterol levels in eggs, and boost antioxidant capacity and immunity in laying ducks. Liu, Huang, et al. investigated the possibility of using a mixture of Chinese herbs as a feed additive in autochthonous (Wenchang) breeder hens, observing that it could improve bird productive parameters (laying rate egg weight, feed efficiency, and egg quality) by enhancing the immune status and anti-apoptosis capacity and reducing potential pathogens in the caecal microbiota, as well as maintaining the intestinal health of the offspring chicks. Another herbal-derived feed additive (jujube leaf extract) was also tested by Kilinç in laying hens, resulting in improved bird productive performance (in terms of increased feed efficiency and egg production), egg quality traits (in terms of higher albumen index, Haugh unit, shell weight, and shell thickness), and slowed down yolk lipid oxidation (in terms of reduced thiobarbituric acid reactive substances levels).

Recent years have seen a rise in interest in the nutritional and therapeutic properties of Chinese yam polysaccharide. The yam's bioactive components were complex, but the water extract's main component, non-starch polysaccharides, gained a lot of interest due to its many potential biological activities (6). Incorporating 0.10 g/kg of Chinese yam polysaccharide copper complex into broiler diets was suggested as an effective way to enhance growth, immunity, and antioxidant capacity. These findings point to the possibility of Chinese yam polysaccharide copper complex as an ecologically friendly feed additive for poultry farms (Zhang J. et al.). *Astragalus membranaceus* is a popular tonic herb in numerous Asian areas. It is known to include components that exhibit diverse biological actions, such as antioxidative, anti-inflammatory, and antiviral characteristics. *Codonopsis pilosula* is recognized for its capacity to improve spleen functionality, support liver wellbeing, and provide anti-tumor, antioxidant, and antibacterial properties. Liu, Xiao et al. looked at how a combination of *Astragalus membranaceus* and *Codonopsis pilosula* extracts affected the health of broiler chickens' intestines, immune system, antioxidant activity, and overall growth performance. The results suggested that broiler chickens' gut microbiota changed in response to dietary CHM, which in turn improved feed utilization, increased mRNA expression of pro-inflammatory cytokines in the jejunal mucosa, and decreased serum endotoxin levels and activities of diamine oxidase and lactate dehydrogenase.

The transcriptome serves as a crucial tool in investigating the kinetics of gene expression, assessing and detecting illness indicators, and expediting the exploration of novel targets. The liver serves as a crucial immunological organ and plays a major role in numerous physiological processes. Hence, employing transcriptomics to investigate the hepatic gene expression in Peking ducks fed silybin-containing diets is valuable for enhancing comprehension of the impact of silybin on the growth and development of Peking ducks. Additionally, it may yield additional insights into the utilization of silybin in animal diets. Zhang Z. et al. found that supplementing Peking ducks with 1,600 mg/kg of silybin significantly improved their growth performance, enhanced their immune capabilities, and reduced their inflammatory responses. The transcriptome analysis indicates that achieving this may involve the regulation of antigen processing and presentation, amino acid metabolism and synthesis, as well as JAK–STAT pathways.

In a study conducted by Suliman et al., the researchers investigated the impact of adding water-based betaine and/or nano-emulsified vegetable oil on the features of carcass and meat quality in broilers that were grown in conditions of heat stress. Water enriched with betaine significantly enhanced carcass dressing weight, breast weight, and meat quality concerning water-holding capability and tenderness during heat stress conditions.

Probiotics, due to their environmentally favorable and non-polluting nature, are commonly administered directly to broilers to suppress the growth of infections and regulate their digestive function (7–9). A common probiotic strain, *Clostridium butyricum*, has remarkable intestinal environment adaptation due to its spores can withstand extreme conditions of temperature, humidity, stomach acid, and bile salts (9). Digestive enzymes found in *C. butyricum* metabolites can reduce the molecular size of nutrients, making them easier for the host to absorb. This improves the nutrients' utilization efficiency, which in turn boosts the host's growth performance (10). Li, Long et al. demonstrated that the incorporation of *C. butyricum* CBM 588 to the diets of broilers enhanced their growth performance and improved the quality of their meat. *C. butyricum* could improve gastrointestinal wellbeing, potentially through the stimulation of the Nrf2 signaling pathway and suppression of the NF-κB signaling pathway. However, further investigation is required to ascertain the precise mechanism by which *C. butyricum* operates.

Paneru et al. suggested that fenugreek seeds, which contain significant amounts of dietary fiber, soluble fiber, and biologically active phytochemicals, could potentially function as a prebiotic. Additionally, they speculated that Fenugreek seeds may have a synergistic effect when combined with *Bacillus*-based direct-fed microbials, leading to health-enhancing advantages and supporting the growth performance of broiler chickens. Results showed that fenugreek seeds and *Bacillus*-based direct-fed microbials affected broiler health and productivity differently at different stages of broiler age. While fenugreek seeds and *Bacillus*-based direct-fed microbials worked together to improve growth performance in the finisher phase, they had opposite effects on gut morphology and blood parameters. To better understand how fenugreek seeds and *Bacillus*-based direct-fed microbials work together, and to find the optimal dose for broiler health and production, the authors suggested the need for more research. Another study was conducted by Azzam et al. to assess the optimal performance-enhancing effects of supplementing laying duck breeders with L-Threonine and *B. subtilis* DSM32315. This study found that supplementing the diet with a combination of L-Threonine and *B. subtilis* DSM 32315 at specific rates (0.7 g/kg for L-Threonine and 0.5 g/kg for *B. subtilis* DSM 32315) had beneficial effects on the eggshell percentage, hatchability, and body weights of newly hatched ducklings. Furthermore, the inclusion of L-Threonine as a separate component at a dosage of 0.7 g/kg has the potential to enhance the egg production of duck breeders.

The shift toward sustainable and eco-friendly practices in poultry feeding, nutrition, and quality is imperative in the post-antibiotic era. This Research Topic explores unconventional solutions, such as the utilization of alternative nutritional strategies, plant extracts, herbal-derived feed additives, and probiotics, showcasing promising avenues to enhance poultry welfare, mitigate antibiotic resistance, and promote environmentally conscious production methods. These innovative approaches not only address the challenges posed by the reduction of antibiotic usage but also pave the way for a more sustainable and resilient future in poultry farming.

## Author contributions

A-MA-M: Supervision, Validation, Visualization, Writing – original draft, Writing – review & editing. IB: Writing – original draft. NM: Writing – original draft. AS: Writing – original draft.
